# The relative efficiency of modular and non-modular networks of different size

**DOI:** 10.1098/rspb.2014.2568

**Published:** 2015-03-07

**Authors:** Colin R. Tosh, Luke McNally

**Affiliations:** 1 School of Biology, Newcastle University, Ridley Building 2, Newcastle upon Tyne NE1 7RU, UK; 2 Centre for Immunity, Infection and Evolution, School of Biological Sciences, University of Edinburgh, Edinburgh EH9 3FL, UK; 3 Institute of Evolutionary Biology, School of Biological Sciences, University of Edinburgh, Edinburgh EH9 3FL, UK

**Keywords:** modularity, evolution, neural network, simulation

## Abstract

Most biological networks are modular but previous work with small model networks has indicated that modularity does not necessarily lead to increased functional efficiency. Most biological networks are large, however, and here we examine the relative functional efficiency of modular and non-modular neural networks at a range of sizes. We conduct a detailed analysis of efficiency in networks of two size classes: ‘small’ and ‘large’, and a less detailed analysis across a range of network sizes. The former analysis reveals that while the modular network is less efficient than one of the two non-modular networks considered when networks are small, it is usually equally or more efficient than both non-modular networks when networks are large. The latter analysis shows that in networks of small to intermediate size, modular networks are much more efficient that non-modular networks of the same (low) connective density. If connective density must be kept low to reduce energy needs for example, this could promote modularity. We have shown how relative functionality/performance scales with network size, but the precise nature of evolutionary relationship between network size and prevalence of modularity will depend on the costs of connectivity.

## 1. Introduction

Modularity in a network of interactions occurs when the network is subdivided into relatively autonomous, internally highly connected components [1]. Modularity is found in diverse systems ranging from an animal's body organ system to protein and other molecular networks. To many non-specialists, the concept of modularity is associated with the brain. Stimulate the base of the forefinger of a monkey and a distinct area of the brain's somatic sensory cortex fires. Move a couple of inches towards the wrist and repeat and a different brain area fires [2]. Information from different areas of the body is processed by different brain areas (note that this is only one of many types of brain modularity). There is a long-running debate starting with Fodor's *The modularity of mind* [3] regarding the extent of brain modularity, how to define modularity, and whether each module should have a discrete function [4]. To many, it will seem obvious that the brain consists of largely independent areas that undertake specific functions but there is also evidence that important, apparently discrete, functions are affected in a shared neural apparatus. People are especially sensitive to faces but training individuals intensively to recognize cars interferes with their ability to recognize faces, indicating that recognition of these objects is not organically distinct [5]. While the present work is informed by and informs this debate, our focus is much narrower and we confine ourselves to the question: why does the structural entity that is a module (as defined above from [1]) appear so frequently in biological networks? What are its benefits and so why has it evolved so frequently?

Much of the computational work on the evolution of modularity had been done using the artificial neural network [6]: an extreme abstraction of cognition that nevertheless displays some properties of real animal cognition [7]. While, strictly speaking, this allies much research on the evolution of modularity to evolution of brain modularity, the neural network formulation has also been used to model gene regulatory networks [8] so insights gained using this model may be more general in application. Indeed the topic of this paper, the issue of networks size in the evolution modularity, has arguably received most attention in non-neural systems such as bacterial metabolic networks [9,10], and we also discuss our results in the context of findings from these systems. Reasonably, it was thought that organization of biological networks into modules is a more efficient way to process information than doing it through non-modular networks. Ultimately, this may be the case but computer simulations do not indicate that the transition from a simple non-modular neural network to a simple modular one necessarily results in more efficient information processing [11]. Computer scientists have, therefore, investigated alternative mechanisms that may promote neural network modularity and these include: node connection costs, synaptic weight noise and modularly varying network goals [12–14]. Numerous other mechanisms are proposed for the evolution of non-neural modularity and many of these mechanisms may also apply to the evolution of brain networks [1].

One factor that has not been investigated exhaustively in previous modelling studies is that of network scale. Most previous studies ([11–14] and see in [11]) have used small neural networks of around 50 nodes or in some cases much fewer. Only one study [15] has systematically considered the issue of network scale in the evolution of modularity. Bullinaria [15] challenged neural networks with a ‘what–where’ task, varied the number of hidden nodes from 9 to 1000, and showed that the architecture evolved through natural selection was always non-modular, regardless of network size. Bullinaria [15] (see also [11]) used a network in which there is no spatial segregation between different inputs on the input layer. An alternative form used in several other studies (e.g. [12–14]) and in this study assumes spatial segregation within the network of different data streams into the network. Here we assume that each input stream is processed by distinct network modules across network layers and all information is integrated late in the information processing sequence. Neural systems of this general form are represented in nature by, for example, the columnar organization of the somatic sensory cortex of mammals [2], the processing of different image attributes within distinct areas of the retina, superior colliculus, lateral geniculate nucleus and early visual cortical areas of primates [16], and the early visual processing apparatus of insects [17].

**Figure 1.**
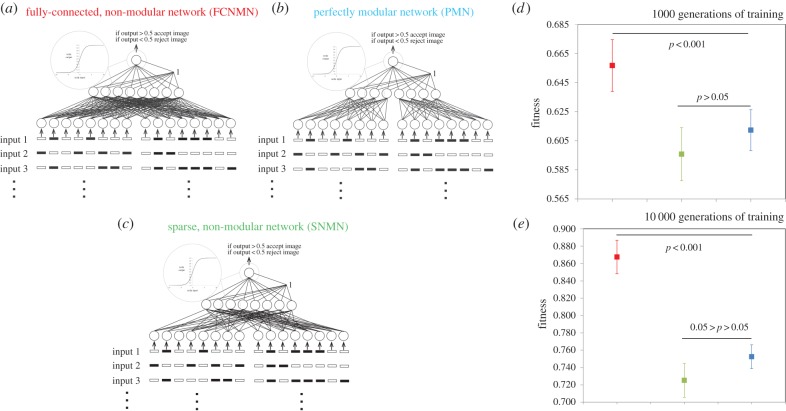
Performance of ‘small’ (*b*) modular (PMN) and (*a*,*c*) non-modular (FCNMN and SNMN) neural networks with the same number of nodes training to identify subsets of random inputs. Mean networks fitness (*n* = 20) with 95% CIs is analysed at generation 1000 and 10 000 of training. Statistics: (*d*) generation 1000; FCNMN (red) versus PMN (blue), *t*_38_ = 4.09; SNMN (green) versus PMN (blue), *t*_38_ = −1.50; (*e*) generation 10 000; FCNMN versus PMN, *t*_38_ = 10.8; SNMN versus PMN, *t*_38_ = −2.39. Note that the vertical axes of plots are not standardized and show different ranges. (Online version in colour.)

**Figure 2.**
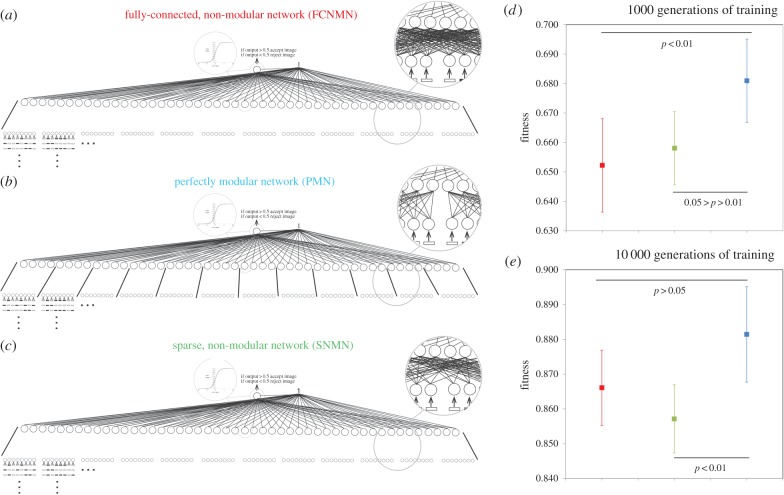
Performance of ‘large’ (*b*) modular (PMN) and (*a*,*c*) non-modular (FCNMN and SNMN) neural networks with the same number of nodes training to identify subsets of random inputs. Mean networks fitness (*n* = 20) with 95% CIs is analysed at generation 1000 and 10 000 of training. Statistics: (*d*) generation 1000; FCNMN (red) versus PMN (blue), *t*_38_ = −2.83; SNMN (green) versus PMN (blue), *t*_38_ = 2.54; (*e*) generation 10 000; FCNMN versus PMN, *t*_38_ = −1.84; SNMN versus PMN, *t*_38_ = 3.01. Refer also to the ‘Material and methods’ section for a complete description of network connectivity. Note that the vertical axes of plots are not standardized and show different ranges. (Online version in colour.)

We present simulations in which the (connective weight) training efficiency and ultimate performance of three types of structurally static neural network architectures, each with the same number of nodes, are investigated: the fully connected, non-modular network (FCNMN); the perfectly modular network (PMN); and the sparse, non-modular network with the same number of node connections as the PMN network (SNMN) (figures 1 and 2). Initially, networks are challenged with the task of identifying a subset of random input patterns from a larger set of random patterns. We compare the performance of ‘small’ (16 input nodes, 8 hidden nodes, 1 output node) networks of the above three types with ‘large’ (96 input nodes, 48 hidden nodes, 1 output node) networks of the same three types. Large modular networks are produced by maintaining module dimensions but increasing module number, with a concurrent increase in the number of data input streams. By varying network input and task, network output encoding and artificial neuron properties, simulations are repeated in four system states to determine the robustness of effects. Additionally, we undertake a less-detailed analysis (less replicated and no sensitivity analysis) of network efficiency across a greater range of network size to inform on the efficiency–size relationship of each network type.

## 2. Material and methods

### (a) System state 1

We ran simulations in four distinct system states to determine how robust the effects are. We describe in detail only system state 1, and changes to this system associated with the other three states are described in the next section.

We used three-layer, feed-forward artificial neural networks with McCulloch–Pitts neurons in the hidden and output layers [6], activation functions of the form: output = 1/(1 + (*e*^-β*input)), *β* = 1, and trainable bias. Input nodes simply received the numerical value of inputs and had weighted connections to the hidden layer. Hidden and output nodes had trainable bias. The connective architecture of networks used is shown in figures 1 and 2. In FCNMNs, each node in the input layer had a weighted connection to each node in the hidden layer. In the PMNs, each module consisted of eight input nodes and four hidden nodes which were full connected but there were no weighted connections between modules. The probability of a weighted connection between the input and hidden layer in the SNMN with *n* nodes was the number of active connection in the PMN with *n* nodes/the number of active connection in the FCNMN with *n* nodes. Only architecture between the input and hidden layers of each network was varied and hidden and output layers were fully connected. Each input set inputting into a single module or equivalent area in non-modular networks was a 256 × 8 array consisting of all binary combinations of eight array elements, positionally randomized with respect to row. The same 256 × 8 × 2 array and 256 × 8 × 12 array input set was used within simulations with the small and large networks, respectively. For each network architecture/network size combination, 20 replicates of 50 networks (the latter is the population size in the genetic training algorithm) were initiated by choosing each connection weight from a uniform random distribution between −3 and 3. Each of the 20 replicates of the small and large SNMNs was also seeded with a different random connective architecture between the input and hidden layer. Each of the 20 replicates of each network size class was allocated a randomly selected subset of 100 inputs from the relevant input sets, and it was the task of the networks within the genetic algorithm to maximize output activity (output > 0.5) in response to these while minimizing response (output < 0.5) in response to the remaining 156 inputs. Formally, fitness was defined as (the number of correct responses i.e. output > 0.5 to the size-100 input subset)/100 × (the number of correct responses i.e. output < 0.5 to the remaining 156 inputs)/156. This gave a metric with a value of 1 when behaviour was perfect and 0 when completely imperfect and some level of performance at both types of task (acceptance and rejection of appropriate subsets) was required for fitness > 0. Note that while each of the 20 replicates was allocated a unique size 100 input set, the same set of 20 was used between network architecture types within network size classes. The genetic training algorithm proceeded by selecting the top performing 10 networks from the 50 in each generation, cloning each of these networks five times, and mutating each weight within these by adding a number selected from a random normal distribution with mean 0 and s.d. 0.25 to form the next generation.

### (b) System state 2

While fundamentally the ‘task’ of the network was changed here this necessarily involved changes to multiple parts of the system and this is desirable as it tests the robustness of effects to major state change. The same input set as system state 1 was employed but now all 1 s within 140 rows of the 256 row input set were converted to a number from a random uniform distribution between 0 and 0.5, with each row receiving a different random number. This procedure was repeated 20 times to produce inputs for the 20 replicates per network architecture type (SNMN, PMN or FCNMN). If one wishes to apply a visual analogy to the input–receiver system; we have modelled, the 116 inputs that are unmodified are visually ‘intense’, whereas the modified inputs are visually ‘dull’. The task of the network was to respond with high output activity to the ‘dull’ inputs (output > 0.5) and to respond with low activity (output < 0.5) to the ‘intense’ inputs. Formally, fitness was defined as (the number of correct responses i.e. output > 0.5 to the size-140 input subset)/140 × (the number of correct responses i.e. output < 0.5 to the remaining 116 inputs)/116. Some aspects of this new input-task system (numbers of inputs in the ‘dull’ subset, decision to ‘accept’ rather than reject dull inputs) are, within bounds, arbitrary and were chosen in some cases simply to ensure the system differed substantially from state 1.

### (c) System state 3

The system was as state 1 but the task of the network was now to respond with an output > 0.9 to the designated size 100 subset of inputs while minimizing response (output < 0.9) in response to the remaining 156 inputs.

### (d) System state 4

The system was as state 1 but *β* of the activation functions of nodes/neurons was set to 0.1. This had the effect of reducing the extent to which node output varied as a result of variation in summed input into the node, i.e. nodes were less ‘sensitive’.

### (e) Analysis

We measured mean fitness with 95% CIs at generation 1000 (a measure which we refer to as ‘speed’ of training for convenience, as it relates the rate at which fitness is gained during the training period in which fitness is gained at a high rate) for each network architecture/network size class combination and again at 10 000 generations by which time most network fitness trajectories had plateaued (we refer to this measure as network ‘ultimate fitness'). The only exception was in system state 2. The task in this system state is easy relative to that in the other system states and networks achieved a high fitness asymptote within 100 generations. We, therefore, quantified performance at 15 and 500 generations of training as measures of ‘speed’ and ‘ultimate performance’, respectively. Variances of datasets to be compared statistically were invariably homogeneous and data were generally normal, so we compared means of the modular network with the non-modular networks within network size classes using *t*-tests. While we have been selective in the comparisons we have undertaken, this still amounts to a considerable number of statistical comparisons across the whole analysis. Comparisons of marginal significance at *α* = 0.05 should be viewed sceptically by the reader. We also quantified the performance of the modular network relative to each of the non-modular networks as effect size, *d*, calculated as eqns 1 and 2 of [18] (figure 6).

### (f) Finer-scale analysis of how network fitness varies with networks size

Networks were conformed as state 1 but their size was varied in numerous steps. Sizes considered were 16/8, 24/12, 48/24, 72/36, 96/48, 120/60, 144/72 (number of nodes in input layer/number in hidden layer, all networks had one output node). Simulations were run exactly as before but using only five replicates per network type/size combination owing to computational constraints.

## 3. Results

**Figure 3.**
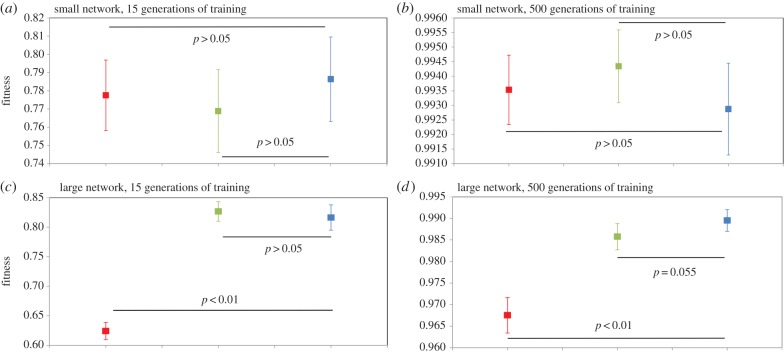
Performance of ‘small’ and ‘large’ modular (PMN) and non-modular (FCNMN and SNMN) neural networks with the same number of nodes training to identify subsets of random inputs. Networks are as those in figures 1 and 2 but now the task of the networks is altered. Now 1 s in 140 of the 256 inputs have a value between 0 and 0.5 and the networks must respond with high output to these and with low output to remaining inputs. Mean networks fitness (*n* = 20) with 95% CIs is analysed at generation 1000 and 10 000 of training. Statistics for small networks: (*a*) generation 15; FCNMN (red) versus PMN (blue), *t*_38_ = 0.784; SNMN (green) versus PMN (blue), *t*_38_ = 1.13; (*b*) generation 500; FCNMN versus PMN, *t*_38_ = −0.700; SNMN versus PMN, *t*_38_ = −1.53. Statistics for large networks: (*c*) generation 15; FCNMN (red) versus PMN (blue), *t*_38_ = 15.5; SNMN (green) versus PMN (blue), *t*_38_ = −0.790; (*d*) generation 500; FCNMN versus PMN, *t*_38_ = 9.51; SNMN versus PMN, *t*_38_ = 1.98. Note that the vertical axes of plots are not standardized and show different ranges. (Online version in colour.)

**Figure 4.**
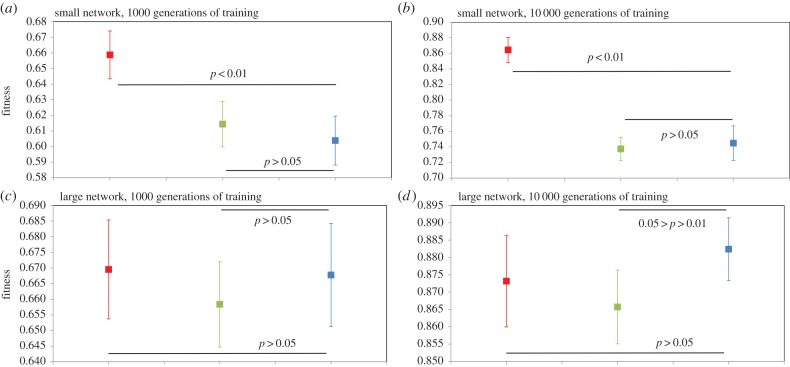
Performance of ‘small’ and ‘large’ modular (PMN) and non-modular (FCNMN and SNMN) neural networks with the same number of nodes training to identify subsets of random inputs. Networks are as those in figures 1 and 2 but now network output encoding is modified. Now networks must respond with an output > 0.9 (rather than > 0.5 as in the networks of figures 1 and 2) to ‘accept’ an input and any output < 0.9 constitutes a ‘rejection’ of that input. Mean networks fitness (*n* = 20) with 95% CIs is analysed at generation 1000 and 10 000 of training. Statistics for small networks: (*a*) generation 1000; FCNMN (red) versus PMN (blue), *t*_38_ = −5.23; SNMN (green) versus PMN (blue), *t*_38_ = −1.03; (*b*) generation10 000; FCNMN versus PMN, *t*_38_ = −9.15; SNMN versus PMN, *t*_38_ = 0.579. Statistics for large networks: (*c*) generation 1000; FCNMN (red) versus PMN (blue), *t*_38_ = −0.162; SNMN (green) versus PMN (blue), *t*_38_ = 0.915; (*d*) generation10 000; FCNMN versus PMN, *t*_38_ = 1.21; SNMN versus PMN, *t*_38_ = 2.49. Note that the vertical axes of plots are not standardized and show different ranges. (Online version in colour.)

**Figure 5.**
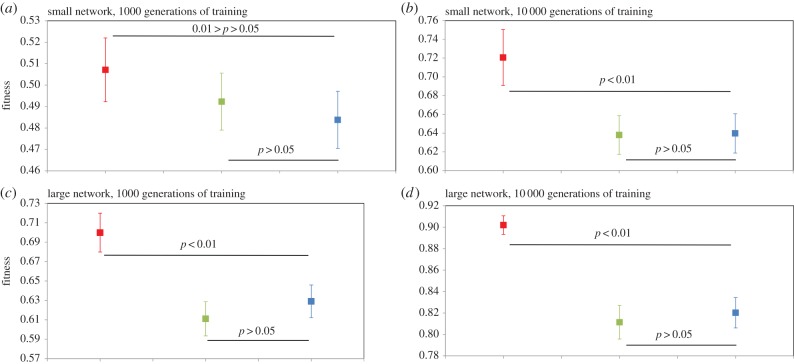
Performance of ‘small’ and ‘large’ modular (PMN) and non-modular (FCNMN and SNMN) neural networks with the same number of nodes training to identify subsets of random inputs. Networks are as those in figures 1 and 2 but now properties of the squashing functions of artificial neurons are modified. Now the *β* parameter in node squashing functions is set to 0.1 (in the networks of figures 1 and 2, the *β* parameter is set to value 1). This modification makes node outputs less sensitive to variation in summed node inputs. Mean networks fitness (*n* = 20) with 95% CIs is analysed at generation 1000 and 10 000 of training. Statistics for small networks: (*a*) generation 1000; FCNMN (red) versus PMN (blue), *t*_38_ = −2.45; SNMN (green) versus PMN (blue), *t*_38_ = −0.876; (*b*) generation 10 000; FCNMN versus PMN, *t*_38_ = −4.66; SNMN versus PMN, *t*_38_ = 0.119. Statistics for large networks: (*c*) generation 1000; FCNMN (red) versus PMN (blue), *t*_38_ = −5.66; SNMN (green) versus PMN (blue), *t*_38_ = 0.132; (*d*) generation 10 000; FCNMN versus PMN, *t*_38_ = −10.3 SNMN versus PMN, *t*_38_ = 0.882. Note that the vertical axes of plots are not standardized and show different ranges. (Online version in colour.)

**Figure 6.**
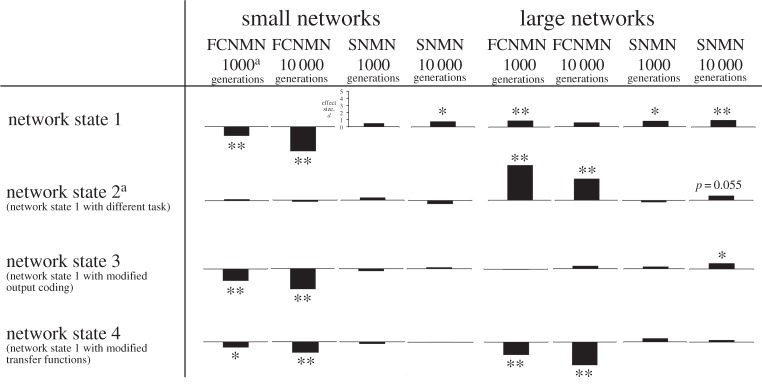
A summary of all data in figures 1–5, with comparisons between the PMN and each of the non-modular networks (FCNMN and SNMN) expressed as an effect size, *d*, calculated as equations 1 and 2 of [18]. Positive effects, in which the modular network performs better that the other network types, are plotted with bar upwards and negative effects are shown with a downwards plotted bar. The statistical significance of effects calculated using the *t*-test is shown above or below bars. * indicates 0.05 < *p* < 0.01, ** indicates *p* < 0.01 and absence of an asterix indicates *p* > 0.05. Statistics associated with these probabilities can be found in the captions of figures 1–5. Superscript a denotes that in network state 2, performance was gauged at 15 and 500 generations rather than 1000 and 10 000 generations because the task was easy and networks achieved high fitness values rapidly. *y*-axis scale, which is the same in each graph, is shown only in one instance.

We have presented all data as means with 95% CI and associated statistical significances in figures 1–5, however, readers may wish to concentrate on figure 6 where all data are presented in a single figure as effect size relative to the modular network. In system states 1–3, there is a pronounced and consistent effect. The performance of the modular PMN relative to the non-modular FCNMN is much greater in large networks compared with small networks. In system state 1, in small networks, PMN performs pronouncedly worse than FCNMN both at 1000 and 10 000 generations. In large networks in systems state 1, however, PMN performs significantly better than FCNMN at 1000 generations and the same as FCNMN at 10 000 generations. In small networks in system state 2, there is no difference between the performance PMN and FCNMN, but in large networks PMN performs better than FCNMN at both 15 and 500 generations of training. In small networks in system state 3, PMN performs significantly worse than FCNMN at both 1000 and 10 000 generations. In large networks, however, there is no significant difference in the performance of these networks.

A much weaker effect in the relative performance of the modular PMN and non-modular SNMN networks is suggested in system states 1–3 (figure 6). Generally, there is no significant difference in the performance of PMN and SNMN in small networks (one exception showing marginal significance in system state 1 at 10 000 generations, figure 6). In large networks, however, performance of the modular PMN is significantly greater than the non-modular SNMN in numerous instances, including: networks state 1, 1000 and 10 000 generations; networks state 2, generation 10 000; networks state 3, generation 10 000. We again emphasize that these effects are not pronounced. For example, effect sizes for PMN versus SNMN in small and large networks are quite similar by eye; it just happens that effect in the large networks cross the *α* = 0.05 threshold on two occasions but only once in the small networks.

None of these effects appear to apply to system state 4 in which neuron properties are changed relative to system state 1. PMN performance is lower than that of FCNMN in small networks at both 1000 and 10 000 generations. In large networks, the same applies but effect size is even greater. There is little difference in the performance of PMN and SNMN in networks of either size.

In summary, network size is an important determinant of the relative performance of modular and non-modular networks in the system studied. In three of the four system states considered, an increase in network size is associated with an increase in the performance of the modular relative to the non-modular networks considered. In the remaining system state, the opposite is true.

**Figure 7.**
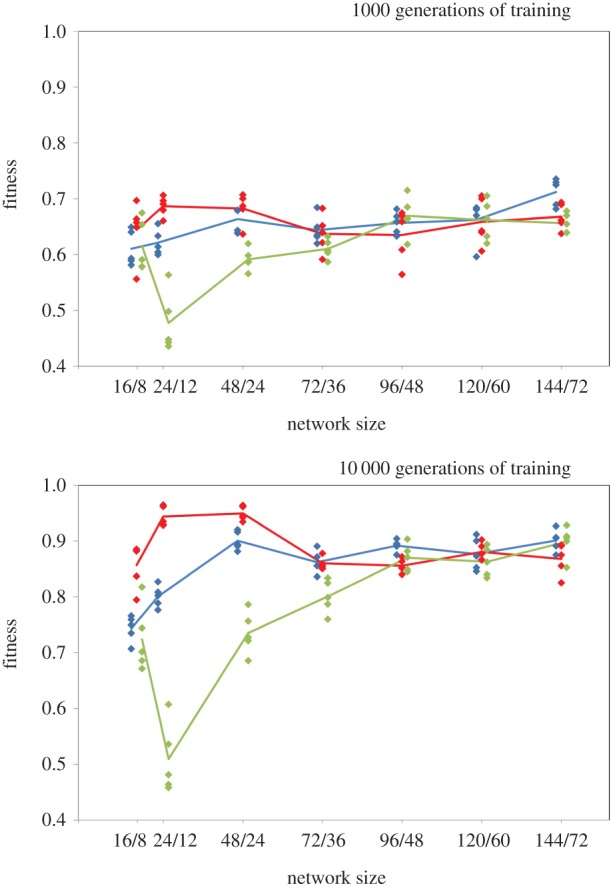
Networks conformed as networks in state 1 (figure 6) with simulations run on networks of different size with a smaller number of replicates at each size. Diamonds are raw fitness values for each simulation run and lines join means of these points. The ratios on the horizontal axis refer to the number of nodes in the input and hidden layers, respectively. All networks had a single output node. Networks of each type (red, blue, green) are offset on the horizontal axis by 0.2 to allow visualization of points. (Online version in colour.)

The finer-scale analysis of network fitness variation with size revealed a wealth of interesting details, however, the most notable effect (an effect not likely be predicted from the coarse-scale analysis above) is that the network efficiency–size relationship of the sparse SNMN differs qualitatively from the FCNMN efficiency–size and PMN efficiency–size dynamic (figure 7). Readers are advised to concentrate on results at 10 000 generations of training as those at 1000 generations are similar but less pronounced. One effect of this difference in the SNMN efficiency–size relation is that in the smaller networks considered here, a perfect modular network is considerably more efficient than a non-modular network with the same number of connections between its input and hidden layer. We cannot confirm that the precise relative efficiencies of the different large networks types analysed in the coarse-scale analysis above (i.e. the PMN networks commonly performing significantly better than the non-modular SNMN and FCNMN) are found in the even larger networks (due to lower replication number), however, there is a general convergence of performance level in very large networks.

## 4. Discussion

We have identified two effects here, that depend on scale of the networks, and that could influence the evolution of modularity in networked systems. Considering first the analysis that is coarse with respect to network size but detailed within each network size (high replication and sensitivity analysis). In the small networks considered, the modular network is inferior in performance to the fully connected non-modular network and usually not significantly different in performance to the sparsely connected non-modular network. In the large networks, the situation is different and the modular network is now commonly superior to both types of non-modular network or if not absolutely superior there is at least an increase in relative performance of the modular relative to the non-modular network. We cannot say whether modular networks are also commonly superior in performance to non-modular ones in even larger networks as the more detailed analysis with respect to network size was less fully replicated owing to computational demands. We can, however, conclude that while certain small non-modular networks substantially outperform the perfectly modular one considered here this does not appear to apply to a range of much larger networks where performance of all networks types appears more similar. In this respect, then, there is likely to be a shift in favour of evolution of modularity in large networks: while modularity is inferior to other strategies in small networks, this does not appear to be the case in large networks.

Considering now the analysis that is detailed with respect to network size but coarse within each network size. Probably the most interesting observation from this analysis was the qualitatively different efficiency–size relationship shown by the connectively sparse networks relative to the other two network types. A consequence of this was that in networks of small-to-intermediate size, sparse non-modular networks with the same number of connections as the modular networks performed dramatically worse than those modular networks. This effect presumably lessens as sparse networks become more connectively dense as fully connected non-modular networks performed better than the modular networks. Applying these results to real biology and in particular real neural networks, in networks within a particular size range (what that range may be in real biological networks is unknown), when the costs of adding connections is significant in the overall energy budget of an organism [19,20] and so connective density should be kept low, there may be a considerable advantage in adopting the modular network conformation in preference to a non-modular one with the same connective density.

Most previous modelling studies using small neural networks have indicated that modularity is not expected to be favoured under a broad range of systems states [11,21,22]. To some extent, this study supports this conclusion. In the small networks, we have modelled there are non-modular networks that are more efficient. However, with variation in network size and the addition of realistic biological assumptions (that connective architecture should be kept low in density for energy efficiency) modularity becomes a more efficient solution. The large networks of the general form we have modelled here in which numerous different input streams are handled by different modules across different layers of neurons are by no means uncommon in nature [2,16,17], and it is tempting to suggest that such systems may have evolved in the first instance (before evolution of alternative functions within such systems) because the modular form is most computationally efficient, without the need for supplementary mechanisms [12–14] (although such additional mechanisms would presumably present further incentive for the evolution of modularity). We do, however, stop short of making this assertion as we have not carried out simulations of structural evolution and it is possible that large networks may have a very pronounced local minimum for non-modularity or that a form of non-modular network intermediate to those we consider here has high fitness. Such simulations are, of course, the next logical step in this research programme investigating the influence of network size on the evolution of modularity. We have only analysed in detail the relative performance of networks of one ‘large’ size, and we do not know if modular networks of even greater size are also commonly more efficient than non-modular networks. Moreover, where we did analyse the performance of large networks in detail, increased relative performance of modular networks with size is not a universal property of the system studies here as it does not hold in one of the four system states considered. It may be that real, large biological networks embody such components that are not conducive to the evolution of modularity. In the remaining three system states, it would be useful to know which aspect of increased scale is responsible for the reduced efficiency of fully connected networks: the increased complexity of the input set, or the very large number of node interconnections. Determination of an exact scale threshold for when large, non-modular networks become less efficient than modular ones and a study of how relative performance varies as networks continue to increase in size would also be welcomed. The results presented here are, nevertheless, striking enough to suggest that authors of many previous studies on the evolution of modularity in neural networks (see Introduction) may wish to return to their simulations to examine the evolution of network form when networks are varied in size from ‘small’ to ‘large’. This work suggests that modularity may become more relatively efficient as networks become ‘large’.

The relationship between network size and number of modules is known for bacterial metabolic networks: number of modules increases with network size, though this increase in modularity with network size plateaus at intermediate network sizes [10]. Many researchers investigating such relationships would probably not consider the ‘function’ of the network or its components as the principal determinant of such a relationship, but instead would focus on the way in which evolutionary novelties are incorporated into the existing network. Thus, Maslov *et al.* [9] took such a structural approach and found that the number of proteins encoded in an organism's genome is expected to increase slower than linearly with the number of metabolic tasks it can accomplish (metabolic modules it has) because as the number of enzymes grows larger, it can re-use its enzymes more often and thus needs to get fewer new ones to master each new task. There are some superficial analogies between the artificial neural network formulation and metabolic function, and we think it worth suggesting that the positive metabolic network size–module number relationship could be because modularity is more efficient in large networks as shown in this study.

The present findings also have technological implications. A common applied use of artificial neural networks is to integrate many complex datasets to assist decision making, for example, in financial prediction where a variety of social and economic predictors may be integrated to inform trader behaviour [23]. If, owing to the nature of input sets, networks become large, users of such networks may wish to consider a modular design. It is also interesting to note that fully connected networks, a common architecture in technological applications of neural networks, trained for 10 000 generations are most efficient at small to intermediate sizes in this study (figure 7).

## Data accessibility

Data available from the Dryad Digital Repository: http://dx.doi.org/doi:10.5061/dryad.g2066.
